# Local Processing Bias Impacts Implicit and Explicit Memory in Autism

**DOI:** 10.3389/fpsyg.2021.622462

**Published:** 2021-04-23

**Authors:** Karine Lebreton, Joëlle Malvy, Laetitia Bon, Alice Hamel-Desbruères, Geoffrey Marcaggi, Patrice Clochon, Fabian Guénolé, Edgar Moussaoui, Dermot M. Bowler, Frédérique Bonnet-Brilhault, Francis Eustache, Jean-Marc Baleyte, Bérengère Guillery-Girard

**Affiliations:** ^1^Normandie Univ, UNICAEN, PSL Université Paris, EPHE, INSERM, U1077, CHU de Caen, GIP Cyceron, Neuropsychologie et Imagerie de la Mémoire Humaine, Caen, France; ^2^UMR 1253, iBrain, INSERM, Université de Tours, Tours, France; ^3^Service de Psychiatrie de l’Enfant et de l’Adolescent, CHU de Caen, Caen, France; ^4^Autism Research Group, City, University of London, London, United Kingdom; ^5^Service de Psychiatrie de l’Enfant et de l’Adolescent, Hôpital Intercommunal de Créteil, Créteil, France

**Keywords:** autism, episodic memory, priming, perception, attention

## Abstract

Autism spectrum disorder (ASD) is characterized by atypical perception, including processing that is biased toward local details rather than global configurations. This bias may impact on memory. The present study examined the effect of this perception on both implicit (Experiment 1) and explicit (Experiment 2) memory in conditions that promote either local or global processing. The first experiment consisted of an object identification priming task using two distinct encoding conditions: one favoring local processing (Local condition) and the other favoring global processing (Global condition) of drawings. The second experiment focused on episodic (explicit) memory with two different cartoon recognition tasks that favored either local (i.e., processing specific details) or a global processing (i.e., processing each cartoon as a whole). In addition, all the participants underwent a general clinical cognitive assessment aimed at documenting their cognitive profile and enabling correlational analyses with experimental memory tasks. Seventeen participants with ASD and 17 typically developing (TD) controls aged from 10 to 16 years participated to the first experiment and 13 ASD matched with 13 TD participants were included for the second experiment. Experiment 1 confirmed the preservation of priming effects in ASD but, unlike the Comparison group, the ASD group did not increase his performance as controls after a globally oriented processing. Experiment 2 revealed that local processing led to difficulties in discriminating lures from targets in a recognition task when both lures and targets shared common details. The correlation analysis revealed that these difficulties were associated with processing speed and inhibition. These preliminary results suggest that natural perceptual processes oriented toward local information in ASD may impact upon their implicit memory by preventing globally oriented processing in time-limited conditions and induce confusion between explicit memories that share common details.

## Introduction

Autism spectrum disorder (ASD) is a neurodevelopmental disorder characterized by atypical visual perceptual abilities with superior performance on many perceptual tasks that require local processing, including the block design task (Caron et al., 2006; Kuo and Eack, 2020), the embedded figures task (Shah and Frith, 1983; Jolliffe and Baron-Cohen, 1997), visual search (Plaisted et al., 1998; O’Riordan et al., 2001) or feature discrimination (O’Riordan and Plaisted, 2001). Several cognitive theories have been proposed to explain this superior visual search for local details. First, the “weak central coherence” account (WCC) developed by Frith and Happé postulates a weakness in integrating local details into a global and coherent form (Happé and Frith, 2006). These authors argue that this is a “cognitive style” rather than a core deficit and that a local processing bias may be overcome when explicit global processing is required. For example, it was found that persons with ASD showed lower levels of global processing on a divided attention task but not on a selective attention task (Plaisted et al., 1999; Van der Hallen et al., 2017), and recently, Avraam et al. (2019) found that, when the local and global levels are not competing, individuals with autism demonstrate robust global organization (grouping processes) that operates even when not directly instructed and questions the WCC theory. The second theory focuses on enhanced low and mid-level processes of perception that allow ASD people to detect and memorize the surface properties of visual and auditory patterns, and is summarized in the Enhanced Perceptual Functioning model (EPF, Mottron et al., 2006). This theory postulates that people with ASD have a natural bias to process stimuli locally confirmed by neuroanatomical and behavioral findings (Mottron et al., 2013a; Chung and Son, 2020). This model also implies that global configurations may be processed in a typical manner when experimental conditions are right, such as when global strategies are more appropriate for performing the task (Mottron et al., 2006). Theses both theories posit that individuals with ASD are biased toward local or featural information rather than global properties of a stimulus. These enhanced perceptual abilities may also result from atypical attentional processes (Plaisted et al., 1999; Keehn et al., 2013; Kaldy et al., 2013). Keehn et al. (2013) reported in Autism an impairment of the three independent attentional networks described in Posner and colleague’s model of attention (Petersen and Posner, 2012): alerting, orienting and executive control networks. Petersen and Posner suggest that impairment of the orienting network and more specifically the resistance to attention disengagement may be at the origin of many behavioral features of ASD. Consequently, visual search superiority may be related to a tendency to over-focus coupled with abnormal attentional disengagement. In this context, the visual local processing bias may result from difficulties in shifting from salient details to the global shape. When such a shift is required, individuals with ASD would need longer to disengage their attention to perform as typical controls.

This atypical perceptual functioning may affect other areas of functioning that people with ASD find difficult such as social interactions or communication (Falter et al., 2012 for ASD people with speech onset delay). Another less investigated domain is memory, which may also be affected by this perceptual bias. An interesting distinction to explore the impact of the perceptual profile of ASD subjects is that between implicit and explicit memory. Implicit memory has been defined as the expression of past experiences occurring beyond the boundaries of consciousness and without any intentional recollection (Graf and Schacter, 1985). Priming is one of the most well-known implicit memory phenomena and refers to a change in the speed or accuracy with which a stimulus is processed following prior experience of the same or a related stimulus. Different kinds of priming have been identified, such as perceptual priming, which is based on the physical properties of the stimuli (Tulving and Schacter, 1990). The few studies that have explored this kind of implicit memory in ASD have shown intact priming effects (Renner et al., 2000; Toichi and Kamio, 2001; Gardiner et al., 2003). These priming tasks provide the opportunity to evaluate the effect of local precedence for the ASD group or global precedence for typically developing participants on memory under conditions that favor automatic processing uncontaminated by conscious attentional processes as well as promoting a participant’s preferred perceptual processing style. Recently, Hine and Tsushima (2018) shown that not explicit but implicit memory was affected by the perception style (perceptual index calculated to the Navon task): local perception style people more greatly used implicit memory than global perception style people.

In contrast to preserved performance in implicit memory, several studies report difficulties in autistic subjects on explicit memory tasks, particularly episodic memory tasks. Episodic memory is defined as the memory of personally experienced events, situated in the temporal-spatial context of their acquisition and which implies “mental time travel” back through one’s past, associated with autonoetic consciousness. Several studies have argued that there is an elaborate encoding deficit in ASD memory and learning as well as retrieval difficulties especially in free recall tasks while cued recall and recognition are mostly preserved (for review see Desaunay et al., 2020). For example, learning of a repeated local context could slow down processing of other trials thereby limiting the integration of these trails into a new context (Kourkoulou et al., 2012). Other studies investigated the effect of atypical perceptual functioning on explicit memory for complex figures and have revealed large variations in performance (e.g., Prior and Hoffmann, 1990). Results often depended on the index chosen to measure the impact of local processing bias on memory; studies that used an index based on accuracy found significantly impaired performance (Minshew and Goldstein, 2001; Kuschner et al., 2009) with no evidence of a local processing bias (Kuschner et al., 2009). Other studies used the Developmental Scoring System developed by Bernstein and Waber (1996) with four parameters: organization, style, accuracy (with two subscores: the main substructures and the details of the complex figure separately), and errors. Using this detailed scoring procedure these studies found that participants used part-oriented strategies on structural elements suggesting local processing (Schlooz et al., 2006; Tsatsanis et al., 2011).

This part-orientated style has to be considered in a developmental context. Several studies using different scoring systems report a developmental shift from part-oriented to a more configurational style (Kuschner et al., 2009; Tsatsanis et al., 2011). For instance in the Tsatsanis et al.’ (2011) study, about 30% young typical children aged from 6 to 14 years preferentially used the part-oriented approach. By contrast, about 10% of the 14–42 years group still used this approach. This percentage contrasts with that of the ASD group where the part-oriented style was present in more than 60% of adults. These results are in accordance with the hypothesis that this “atypical” performance may reflect a delay in development in global/local visual perception in ASD related to maturation of brain connectivity (Kuschner et al., 2009; Crespi, 2013).

Studies of the impact of local processing bias have tended to focus on true memory rates without taking into account false positive errors. Moreover, they have tended to use complex figures as stimuli. These might not be the most appropriate stimuli to address the impact of local processing bias because they provide no data either on intrusions or on false recognitions. By contrast, studies published using verbal learning tasks generate both these measures (Minshew and Goldstein, 1993; Bennetto et al., 1996; Bowler et al., 2000). Other studies have used verbal false memory tasks derived from Roediger and McDermott’s paradigm and found contradictory results (Beversdorf et al., 2000; Bowler et al., 2000; Kamio and Toichi, 2007). These contradictions could result from the fact that the impact of processing bias for local information, as described by Frith and Happé, on language is now being debated. This bias would be preferentially observed for non-verbal tasks. Only one used geometric figures with associated visual lures (Hillier et al., 2007). In that study, participants with ASD were better at discriminating true items from false items compared to typically developed comparison participants. We could speculate that if ASD participants had been instructed to process specific details inserted at the same place as the lure items, they would have been more likely than comparison participants to falsely recognize the lures.

The present study aimed to examine the impact of a local bias on both implicit and explicit memory under conditions that promote either local or a global processing. For our implicit memory task, we hypothesize that in the condition favoring local precedence participants with ASD would perform significantly better compared to comparison participants. We predict the reverse pattern in the global condition, where the typical global precedence would favor the comparison participants. In contrast to implicit memory tasks, explicit memory tasks favor conscious mechanisms and globally oriented processing when ASD participants are instructed to focus on the whole of the target. We predicted that in the global condition, participants with ASD would not differ from comparison participants, whilst local processing would increase confusion between targets and lures that share the same details during the recognition test. We tested these hypotheses by conducting two experiments using implicit and explicit memory paradigms. In order to study these effects more in depth and elaborate our cognitive hypothesis, we conducted additional exploratory analyses with more general cognitive functions including local/global precedence, working memory, executive functions, and episodic memory. All these functions were evaluated with standard tests and scores were correlated with those of the experimental tasks.

## Materials and Methods

### Participants

Seventeen participants with ASD and 17 typically developing (TD) comparison participants aged from 10 to 16 years were included in the present study (Table 1). The recruitment started prior to the 2013 publication of DSM5, hence participants had all been diagnosed with verbally and intellectually high-functioning autism or Asperger’s syndrome according to DSM-IV (American Psychiatric Association, 2000) criteria. The diagnosis was established by experienced professionals using the Autism Diagnostic Interview-Revised (ADI-R; Lord et al., 1994) and/or Autism Diagnostic Observation Schedule (ADOS; Lord et al., 1989). The comparison group was recruited among several French schools. Exclusion criteria for both groups were as follows: history of previous neurological disease (other than ASD in the clinical group), head trauma, current psychoactive medication, intellectual disability, and learning disabilities. Families were given a comprehensive description of the research. Requirements of the local Ethical Committee were met and we obtained written consent from parents of minors, in line with the guidelines of the relevant ethics committees.

**TABLE 1:** Participant characteristics (means and standard deviations, SD), and analyses for group differences (independent samples *t*-tests).

	**ASD (*N* = 17)**		**Comparison (*N* = 17)**		**Group differences p and effect size**
	**Mean**	***SD***	**Mean**	***SD***	
Age (in months)	161.87	26.29	161.78	19.99	*ns, r* = 0.002
**Wechsler Intelligence Scale**					
Verbal Comprehension Index	100.50	18.70	111.73	11.01	*ns, r* = 0.34
Perceptual Reasoning Index	101.18	14.67	103.93	10.58	*ns, r* = 0.11
Processing Speed Index	88.25	16.36	102.27	11.63	*p* < 0.05, *r* = 0.45
Working Memory Index	94.00	16.86	106.60	12.01	*p* < 0.05, *r* = 0.40
Spatial span	4.00	0.63	5.07	1.03	*p* < 0.005, *r* = 0.54
**Executive functions**					
Inhibition	20.62	9.39	25.20	6.23	*ns, r* = 0.28
Planning (1st trial)	7.31	1.35	7.40	1.50	*ns, r* = 0.03
Semantic Fluency	29.00	12.80	31.94	5.29	*ns, r* = 0.15
Phonemic Fluency	15.12	5.75	17.47	4.31	*ns, r* = 0.22
**Episodic memory**					
Immediate story recall	19.56	11.13	27.80	8.06	*p* < 0.05, *r* = 0.40
Delayed story recall	17.31	11.76	26.47	7.94	*p* < 0.05, *r* = 0.42
Story recognition	12.00	2.42	13.53	1.36	*p* < 0.05, *r* = 0.37
Rey recall	17.78	7.40	21.80	5.29	*ns, r* = 0.30
**Perceptual bias**					
Local precedence	5.28	4.92	1.37	4.69	*p* < 0.05, *r* = 0.36
Global precedence	0.71	3.73	3.51	5.39	*ns, r* = 0.29

**ns*, non significant.*

### General Cognitive Assessment

All the participants underwent a general clinical cognitive assessment including IQ, working memory, executive functions, episodic memory and local/global precedence aimed at documenting their cognitive profile and enabling correlation analyses with experimental memory tasks. Participants’ IQ was assessed using the Wechsler Intelligence Scale for Children (WISC-IV, Wechsler, 2005). Groups were matched for age, gender, the Verbal Comprehension Index and the Perceptual Reasoning Index (Table 1). The ASD group differed from the comparison group for the two other Wechsler’s indices, i.e., Processing Speed Index and Working Memory Index. ASD participants scored poorly on spatial working memory measured with the spatial span task (Farrell Pagulayan et al., 2006). This task is similar to the classical Corsi Block Tapping Task but we calculated a basal score representing the highest level at which the participant correctly reproduced the four sequences. Executive functions were in normal range. These included inhibition (interference score on the Stroop task, Albaret and Migliore, 1999), planning capacities (number of problems correctly solved at the first trial of the Tower of London, Lussier et al., 1998) and strategies of retrieval from semantic memory assessed with two fluency tasks (Cardebat et al., 1990), i.e., semantic (name of animals) and phonemic (words beginning by the letter *p*) fluency tasks. Episodic memory was assessed by means of the story recall task (CMS) and the Rey-Osterrieth Complex Figure Test. ASD participants obtained pathological scores in the story recall task (*p* < 0.05). Finally, local and global precedence were investigated with a selective attention task used in the Plaisted et al. (1999) study and adapted from the Navon task (Navon, 1977). Briefly, the participants were presented large letter shapes made up of smaller letters that were either the same as or different from the larger letter. They were asked to process either the large (global condition) or the small letter (local condition) in two sessions where they had to identify either the small letter or the large letter in the presented stimuli. Target letters were “H” and “S.” Three kinds of stimuli were provided: compatible stimuli where the large and small letters were the same (S/S and H/H), incompatible where the large letter and the small letter were different (H/S), and neutral stimuli which corresponded to either an “H” or an “S” at a global level when participants had to judge the large letter (a large H made up of small As, and a large S made up of small As) or at a local level when they were required to judge the small letter (a large A made up of small Hs and a large A made up of small Ss, see Plaisted et al., 1999 for methodological details). We calculated two *precedence indices*, one for each condition consisting of the advantage of the compatible trials compared to the neutral. ASD participants differed significantly from the comparison group only for the local precedence index.

The age-related effects on performance were analyzed by means of Pearson correlation coefficients. No significant correlation was obtained in the ASD group. On contrary we observed a significant increase in performance with age in the comparison group for inhibition (*p* = 0.001), planning (*p* = 0.05), and retrieval strategies assed with the semantic verbal fluency task (*p* = 0.001).

## Experiment 1

The first experiment focused on the effect of perceptual bias on implicit memory. The priming task consisted of tachistoscopic identification of drawings of common objects using two distinct encoding conditions: one favoring local processing (Local condition) and the other favoring global processing (Global condition) of drawings.

### Participants

All participants took part in this first experiment. For those who participated in both Experiments 1 and 2, the priming task was always conducted first to avoid the interference from the intentional memory strategies on the implicit memory task.

### Stimuli

A customized database of 220 colored drawings, divided into 20 semantic categories of living and non-living common objects, was created by a cartoonist. On the basis of a pre-experimental pilot study (200 subjects aged 10–20 years), we selected 160 drawings, which were always successfully identified in under 160 ms, the time limit used in the tachitoscopic task. These 160 items were then divided into 16 lists of 10 items. We ensured that the 10 items of one list belonged to different semantic categories and verified that each list yielded a 50% rate of “yes” responses during the study phase for both local processing (containing a small circle in a particular part of the object) and global processing (the global size of the object that could fit into a square of 10 cm width). To avoid potential item effects, we created 60 combinations, each comprising of six lists of target items shown at both study and test phases, four lists of distractors which were provided during the study phase only and six lists of control items, provided during the test phase only.

### Procedure

**FIGURE 1:**
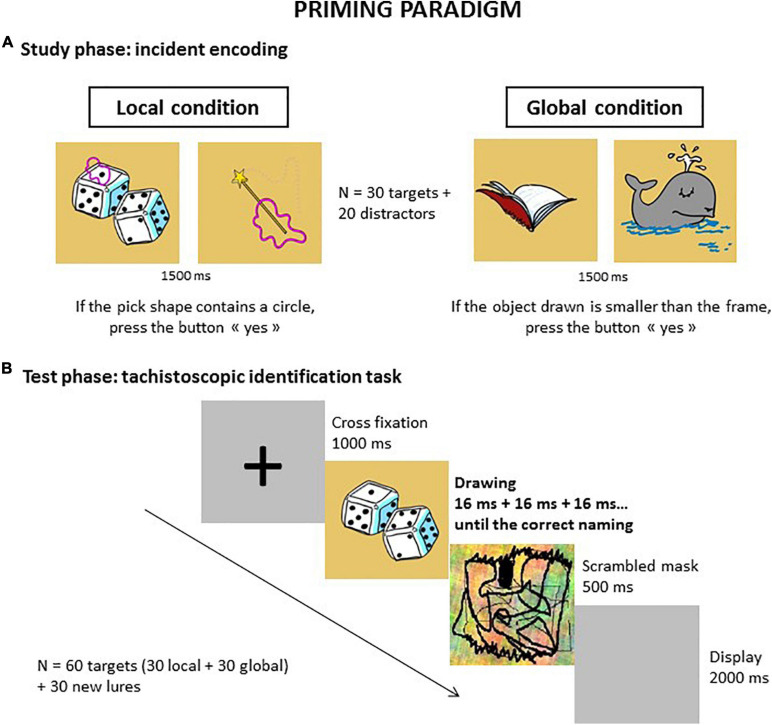
Priming paradigm. The task included a study phase with both local and global conditions provided in a counterbalanced order between subjects **(A)**, followed after a 10 min delay by a test phase **(B)** which consisted in identifying all studied drawings presented with an initial 16 ms time duration and increased with an incremental step of 16 ms up to correct naming.

Participants were placed in front of a 17-inch laptop screen a in a quiet room. They were shown the different series of drawings using E-prime. The task was divided into a study phase containing both local and global conditions, in counterbalanced order between the subjects, and a test phase with all the studied drawings. The study phase consisted of showing 50 drawings per condition: 30 targets and 20 distractors. Each trial started with a fixation cross placed in the center of the screen for 1000 ms, followed by a drawing presented for 1500 ms and a gray display that disappeared either when the participant responded or when 5000 ms had elapsed. The instructions were different in the Local and the Global conditions. In the Local condition, each drawing contained a pink shape on a small part of the object and the participant had to decide if there was a dot in this shape (Figure 1). In the Global condition, an empty frame was provided as a reference measurement to judge if each object drawn was smaller or larger than the frame. Participants were encouraged to process each object globally when performing the task. They responded by pressing one of two keys on a response box. A training phase was provided to ensure that all participants had understood the instructions. During this study, we collected both accuracy scores and response times. After 10 min delay, filled by the Stroop task, the test phase began. This phase was described as a new different task and consisted of a tachitoscopic identification task containing the 60 targets (30 local + 30 global) and 60 new lures, i.e., the non-studied drawings. Each trial started with a fixation cross during 1000 ms followed by a drawing, a scrambled mask specific to each drawing respecting the same perceptual properties as the drawing and designed to limit the persistence of vision and finally, another gray display. The duration of presentation increased until the participant named the object: the first duration was 16ms with an incremental step of 16 ms up to 160 ms maximum. Two breaks of 30 s maximum were introduced during this test phase. The experimenter recorded the number of presentations needed to give the correct answer. As is usual in neuropsychological research of this kind (see Roediger and McDermott, 1993), we calculated a priming index for each experimental condition by subtracting the number of exposures for targets, either local or global, from the number of exposures for new lures. The priming effect is revealed by a significant difference between the two types of items and by a positive index.

### Statistical Analyses

Statistical analyses were conducted using Statistica software. We ran analyses of variance (ANOVAs) using a General Linear Model procedure on response time, accuracy and priming scores. We also calculated effect sizes (η^2^ or r according to the test used). *Post hoc* multiple comparisons were Tukey-corrected. We also conducted Pearson correlations to test the possible association between age and behavioral performance and the relation between other cognitive functions and priming scores in both groups.

### Results

#### Study Phase

**FIGURE 2:**
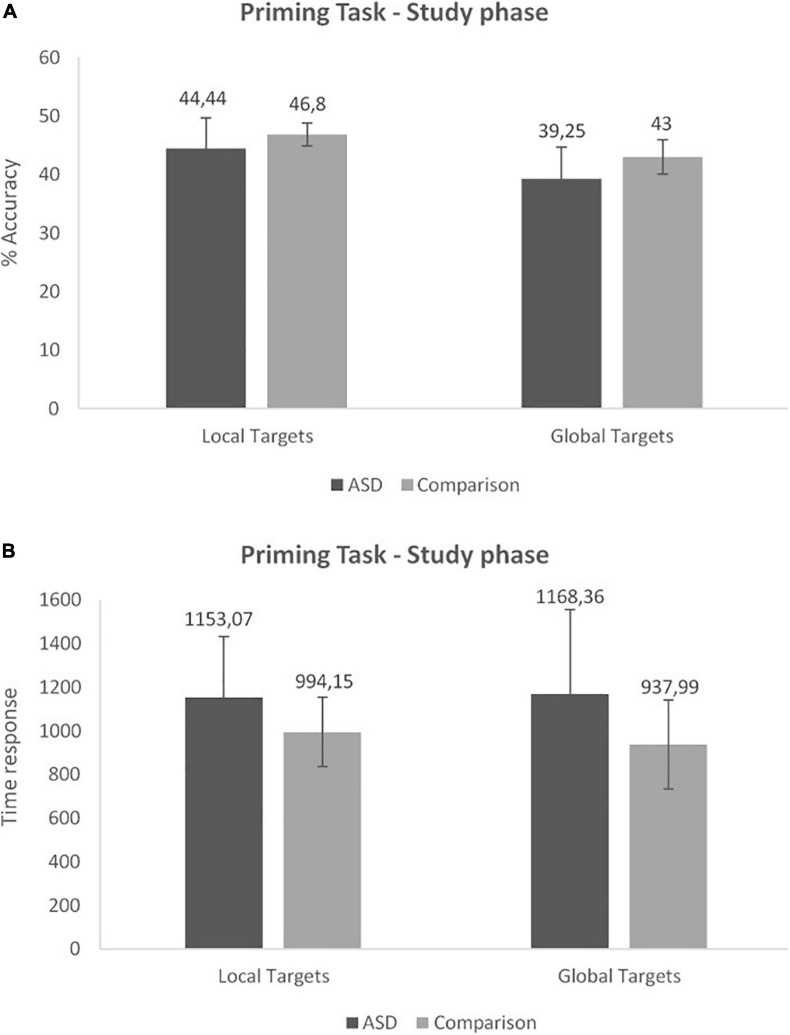
Priming task: accuracy **(A)** and time response **(B)** at the study phase (Mean and SD).

The 2 (Group) × 2 (Condition) repeated measures ANOVA conducted on response time at the study phase revealed a significant effect of Group [*F*(1,29) = 4.42, *p* = 0.04, $\eta_{p}^{2}=0.13$]. The ASD group was slower than the Comparison group in both conditions (Figure 1). No other significant effect or interaction was found (Figure 2B).

The 2 (Group) × 2 (Condition) repeated measures ANOVA conducted on accuracy at the study phase revealed a significant effect of Group [*F*(1,29) = 5.62, *p* = 0.02, $\eta_{p}^{2}=0.16$]. The ASD group performed significantly worse than the Comparison group. We also observed a significant effect of Condition [*F*(1,29) = 33.65, *p* < 0.001, $\eta_{p}^{2}=0.54$] with no interaction between factors. Performance on the Local condition was higher than on the Global condition in both groups (Figure 2A).

#### Test Phase

**FIGURE 3:**
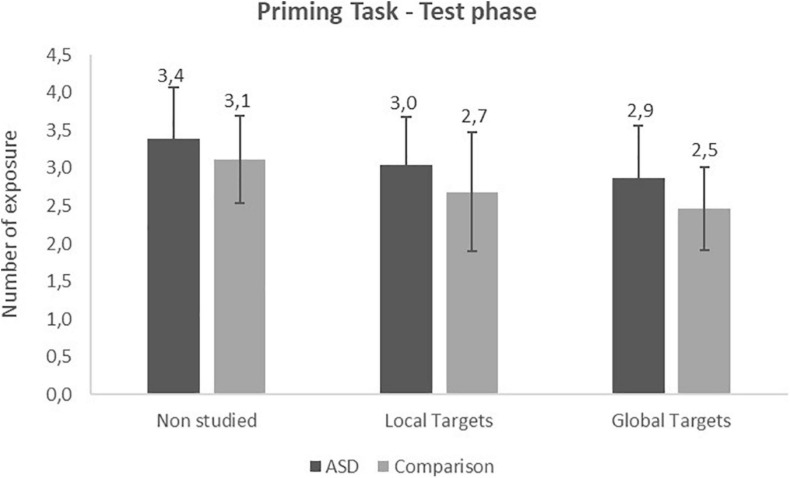
Priming task: number of exposure at the test phase (Mean and SD).

The 2 (Group) × 3 (Items: local target, global target, non-studied) repeated measures ANOVA performed on the number of exposures at test revealed a significant effect of Items [*F*(2,58) = 32.74, *p* < 0.001,$\eta_{p}^{2}=0.53$]. Non-studied items needed more exposures in order to be identified than local (*p* < 0.001) and global targets (*p* < 0.001). Hence, we observed a significant priming effect for local and global conditions (Figure 3). In addition, global targets were identified faster than local ones (*p* = 0.01). There was no other significant effect or interaction. However, we noticed that the difference observed between local and global target in the comparison group (*p* = 0.04) was not found in the ASD group (*p* = 0.1).

According to these results, the 2 (Group) × 2 (Priming index) repeated measures ANOVA confirmed the previous results showed a significant effect of priming index [*F*(1,29) = 5.98, *p* = 0.02, $\eta_{p}^{2}=0.17$] with greater priming in the Global condition compared to the Local condition.

### Age-Related Effect on Performances

Pearson analyses revealed no significant correlation between age and any scores except for Global Priming index in the Comparison group (*r* = −0.57, *p* = 0.03) where the magnitude of priming decreased with age.

### Relation Between General Cognitive Function and Priming Indices

We conducted exploratory correlational analyses between cognitive functions and the two priming indices calculated for each condition to identify possible contribution of some other specific functions to the priming effects. We obtained some significant correlations but only one survived Bonferroni correction. However, we preferred to report these non-significant correlations after Bonferroni correction (NS) to get an overview of the possible cognitive mechanisms associated with priming in each group. The only significant correlation obtained in the ASD group was the positive association between the global priming index and the phonemic fluency (*r* = 0.51, *p* = 0.04, NS). We observed the reverse association in the Comparison group: the global priming index was negatively correlated with performance on semantic fluency (*r* = −0.87, *p* = 0.03, NS), planning (*r* = −0.75, *p* = 0.001) and inhibition (*r* = −0.50, *p* = 0.05, NS). Finally, in the Comparison group, only local precedence correlated significantly with the local priming index (*r* = −0.56, *p* = 0.04, NS), where Local Priming increased when local precedence diminished. In other words, this result could reflect the need to go beyond local perception in order to perform the tachitoscopic identification task.

### Discussion

The main objective of this experiment was to identify the effect of local or global oriented processing on implicit memory using a tachitoscopic identification task. During the study phase, the ASD participants were less accurate and slower than the comparison group. This slowness is corroborated by the processing speed index derived from the Wechsler test. This characteristic is a well-known feature of autism and is also observed in younger populations (Oliveras-Rentas et al., 2012; Hedvall et al., 2013). Concerning accuracy, participants with ASD made more errors than comparison participants that may reflect greater difficulty making these perceptual judgments. However, the difference in performance between the two experimental conditions is similar to that of the comparison participants. Both groups of participants were less accurate in processing the size of the whole target relative to the standard-sized frame placed beside the computer (Global condition) than they were at processing a dot (Local condition). Beyond these differences in complexity, we observed a significant priming effect in both local and global conditions in both groups. These results are in accordance with previous published priming data collected with different experimental paradigms (Gardiner et al., 2003).

Contrary to our expectation, the analysis of the priming index showed no superiority for the Local condition in the ASD group. Instead, we observed a significant advantage for the Global condition, which was reduced for the ASD participants. When we considered each group separately, we found this advantage only in the Comparison group. These results confirm our second hypothesis that predicts better performance in the global condition only in the comparison group. This may be because participants with ASD naturally and automatically process details first and consciously extend to the whole drawing in a second step. Note that this improvement remains discrete because local bias may not present be among all ASD participants. The tachitoscopic presentation may constrain ASD participants to a local processing style by not giving them enough time to shift from salient details to the whole shape. This interpretation is in accordance with the results of the correlation analyses. Whereas the control functions tested by the fluency task were implicated in the Global priming of the ASD group, they were not in the Comparison group. In addition, our data showed that local precedence may have had a negative influence on identification performance during tachitoscopic presentation. Taken together, our results suggest that natural perceptual processes oriented toward local information, associated with a lack of attentional disengagement to perform a global oriented processing, in ASD impact upon their implicit memory by preventing global processing in time-limited conditions.

## Experiment 2

This experiment aimed at identifying the impact of the perceptual processing bias on episodic memory. We pursued this question by providing two different recognition tasks of cartoon pictures that favored either local or a global processing. During the “local condition,” participants had both to process a specific detail and remember each cartoon followed by a recognition task where targets were mixed with two kinds of lures: half with the same details as those of the target and half with new, different lures. The purpose of this design was to reveal a local memory bias resulting in an increase in confusion between targets and lures when they shared the same details. We expected that this local bias would be more likely in the ASD group. In contrast, the “global condition” consisted of processing each cartoon as a whole by making an indoor/outdoor judgment. As in the previous condition, the recognition task contained two kinds of lures, half with the same background as that of the target and half with new, different lures. An increase in confusion between targets and lures sharing the same background would provide evidence of a global bias in perceptual processing. We expected this pattern to be more evident in the control group.

### Participants

Among the children and adolescents who participated in the first experiment, 13 ASD participants and 13 TD participants were tested here [age range: 79–180 months (6.6–15 years), mean = 135.77 ± 28.19 months (11.31 ± 2.35 years)]. All children and adolescents took part in both experiments on the same day. The remaining participants were given another and more difficult version of the episodic memory task that mixed the two conditions, *i.e.*, local and global, in a same test. We observed a floor effect in the first participants, which led us to make the present methodological adjustments.

### Stimuli

**FIGURE 4:**
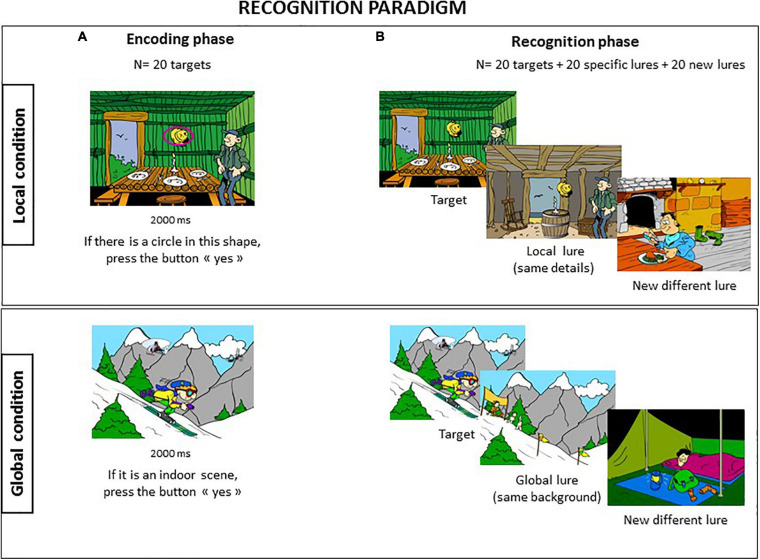
Recognition paradigm. The recognition task was divided into two separate conditions [**(A)** Local and **(B)** Global], provided in a counterbalanced order between subjects, and each involved an incident encoding phase followed after 10 min delay by a yes/no recognition task.

Two tasks were created; each consisted of 60 cartoon pictures with the same visuo-spatial structure drawn by the same cartoonist as drew the pictures for Experiment 1. The stimuli included an equal number of indoor and outdoor situations. Each task contained a set of 60 cartoons that were divided as follows: 20 targets, 20 specific lures oriented toward either “local” (local condition) or “global” (global condition) properties of targets, and 20 different lures (Figure 4).

### Procedures

#### Local Condition

During the intentional encoding phase, participants were shown 20 cartoon pictures, each containing an object surrounded by a pink mark and presented for 2000 ms. The participants were requested to look for a specific detail, i.e., a circle inside the mark, and provide a response as soon as possible by pressing the “yes” or “no” button on a response pad. There was 50% of probability of finding a circle on these 20 targets. They had also to remember the scene. After a 10-min delay, the recognition phase was conducted where the 20 targets were mixed with 40 lures. There were 20 “local” lures that contained the same details located in the same place as the 20 targets, i.e., a “local” lure for each target, and 20 totally different new lures. Participants were asked to discriminate the targets from lures.

#### Global Condition

This condition followed the same procedure as the local condition. The task was composed of an intentional encoding phase where 20 targets were provided with an indoor/outdoor judgment for each cartoon picture. After 10 min, the recognition phase was conducted with the 20 targets mixed with 20 “global” lures characterized by the same background as the 10 targets (five indoor and five outdoor situations) and 20 other different lures.

For each condition, we calculated three scores: a discrimination index (*d*’ or *d*-prime), proportion of Hits, the proportion of false recognitions for specific lures, i.e., local or global, and for the new different lures. The discrimination index takes into account Hits and False Recognitions for specific lures, i.e., “local” for local condition and “global” for global condition.

### Statistical Analyses

Statistical analyses were conducted using Statistica software. We performed independent samples *t*-tests and calculated effect sizes (*r*). We also conducted Pearson correlations to test the possible association between age and behavioral performance and the relation between other cognitive functions and episodic scores in both groups.

### Results

The ASD group differed significantly from the comparison group only on the *d*’ index in the local condition (Table 2) with the ASD group scoring lower than the Comparison group (*p* < 0.05; *r* = 0.40).

**TABLE 2:** Performance at the recognition task.

	**ASD**		**Comparison**	
	**Mean**	***SD***	**Mean**	***SD***
**Local condition**				
Discrimination index (*d*’)*	0.84	0.58	1.39	0.68
Hits	0.55	0.18	0.62	0.15
False recognitions “local”	0.26	0.14	0.17	0.12
False recognitions “new”	0.08	0.13	0.02	0.04
**Global condition**				
Discrimination index (*d*’)	2.98	2.51	2.35	1.41
Hits	0.80	0.17	0.79	0.12
False recognitions “global”	0.17	0.23	0.13	0.08
False recognitions “new”	0.12	0.17	0.08	0.10

*p < 0.05; *r* = 0.40.

### Age-Related Effect on Episodic Memory Scores

Pearson analyses revealed no significant correlations between age and the memory scores (max. *r* = −0.44, *p* = 0.17) except for False Recognitions of local lures in the Comparison group (*r* = 0.67, *p* = 0.02, NS).

### Relation Between General Cognitive Function and Episodic Memory

As for Experiment 1, we conducted exploratory correlation analyses between cognitive functions and the discrimination index and local/global false recognitions (Table 3). For the ASD group, these analyses revealed significant positive correlations between *d*’ in the local condition and the Processing Speed Index, inhibition, and recognition performance in the story recall task. False Recognitions of local lures were negatively correlated with the inhibition process (*p* < 0.001). Other negative correlations were observed in the global condition between False Recognitions of global lures and the Verbal Comprehension Index, planning, and recognition performance in the story recall task (*p* = 0.001). These are interesting but preliminary results that did not survive Bonferroni correction except two of them related to False Recognitions (*p* < 0.001 and *p* = 0.001).

**TABLE 3:** Significant correlations (and *p*) between general cognitive function and episodic memory scores in the ASD group.

	**ASD**			
	***Local condition***		***Global condition***	
	***d*’ index**	**FR “local”**	***d*’ index**	**FR “global”**
**Wechsler Intelligence Scale**				
VCI				−0.62 (0.03)
PRI				
PSI	0.63 (0.03)			
WMI				
Spatial span				
**Executive functions**				
Inhibition	0.60 (0.04)	−0.87 (0.0001)		
Planning (1st trial)				−0.63 (0.03)
Semantic Fluency	0.64 (0.02)			−0.60 (0.04)
Phonemic Fluency				
**Episodic Memory**				
Immediate story recall				
Delayed story recall				
Story recognition	0.64 (0.03)			−0.84 (0.001)
Rey recall				
**Perceptual bias**				
Local precedence				
Global precedence				

*VCI, Verbal Comprehension Index; PRI, Perceptual Reasoning Index; PSI, Processing Speed Index; WMI, Working Memory Index; FR, False Recognition.*

For the comparison group, a significant negative correlation was found between *d*’ in the global condition and Rey recall performance (*r* = −0.66, *p* = 0.03, NS).

### Discussion

The main objective of this second experiment was to test the possible confusion between targets and lures on the basis of shared local features in participants with ASD. We hypothesized that false recognitions would be higher in the ASD group compared to comparison participants in the Local condition. The data collected confirm this hypothesis by showing that the ASD group had difficulties in discriminating lures from targets when they shared common details. The correlation analysis revealed that these difficulties were associated with processing speed and inhibition. We did not observe any difference between groups in the Global condition but correlation analysis showed that the capacity to reject lures is associated with a more general index of verbal comprehension and recognition memory.

Detail-oriented processing at study reinforces ASD participants’ natural preference for local precedence and interferes with recognition judgments. This is not overcome when intentional learning is requested. This result both confirms and extends studies on recognition memory using a recognition index that combined correct and false recognitions (Bowler et al., 2000) by limiting this confusion to experimental conditions that promote local processing. Interestingly, this phenomenon seems to be associated with the capacity to inhibit inappropriate but salient details common to target and lure. A relation with effortful or executive functions and memory has been previously reported using relational memory tasks (Maister et al., 2013). In addition, correlation between the discrimination index and processing speed would appear to confirm this hypothesis. In contrast, recognition based on a globally oriented processing is correlated with a more general capacity of verbal comprehension. Picture encoding in memory relies on both verbal and visual coding (Hockley, 2008) and difficulties in verbal comprehension may share common mechanisms with global integration of the scene.

We did not observe the same influence of executive functions within the Comparison group; instead, we found a dissociation between the capacity to recall a complex detailed figure and correct discrimination after global processing. Hence, in typically developing people, global and local memory based processing may be two more independent mechanisms.

## General Discussion

The present pair of experiments investigated the impact of a local bias observed in ASD on both implicit and explicit memory in conditions that promote either local or global processing. Experiment 1 confirmed earlier findings of preserved priming effects in ASD. Overall, both groups of participants showed the same pattern of performance with slight modifications. Our findings showed that participants with ASD seem less advantaged by the Global condition than were the TD group. Experiment 2 focused on explicit memory and revealed a slight but significant difference in the capacity to discriminate lures from targets in the Local condition. Participants with ASD were less able to discriminate targets from lures than were TD participants when the targets and lures shared the same details. Taken as a whole, the data suggest ASD-related difficulties in consciously inhibiting details. Our overall results confirm our hypotheses that the presence of a local bias in ASD may interfere with both implicit and explicit memory processing.

### Cognitive Profile of the ASD Group

The two groups of participants were matched on age, gender, verbal comprehension index and perceptual reasoning index. However, the clinical and complementary measures revealed that the ASD group performed significantly worse than the comparison group on processing speed and working memory tasks. The additional assessment of working memory using a spatial span task confirmed this result. These findings are in accordance with the profile published by Mayes and Calhoun (2008). Other findings in the present study suggest that the impairment in long term memory is limited to story recall and recognition. Stories are thought to be instances of complex material that require strategies based on verbal cues in order to understand the whole story. ASD individuals are well known to experience greater difficulties with increasing complexity of the material (Minshew and Goldstein, 2001). The final difference observed between groups concerns local precedence: the ASD group in the present study is characterized by a greater local precedence than TD individuals. The presence of this local bias despite the great variability observed in our groups provides the starting point from which to discuss the impact of local processing bias on the memory performance of our participants.

### Impact of Perceptual Bias on Memory

The present study reveals that the part-oriented processing that characterizes ASD may influence both implicit and explicit memory performance. This automatic bias may limit global processing in time-limited conditions as in the present study where tachitoscopic presentations were used. This may have contributed to the overall slowness observed during experimental tasks (Williams et al., 2013) and which is reported by parents in everyday situations that require global processing, and is in line with the arguments of Van der Hallen et al. (2015). In addition, this bias may induce confusion between memories that share common details as demonstrated in the present investigation. This result contrasts with Hillier et al.’s (2007) study which found no difference with a visual paradigm. By contrast, the objective of the task developed for the present study was to test the impact of locally oriented processing on memory using targets and lures that shared strictly similar details for processing during the study phase. Focussing on these details led individuals with ASD to falsely recognize corresponding lures. This result may help to further explain findings from other areas of research, such as Maras and Bowler’s finding of lower ASD-related accuracy and higher recall of incorrect details in their eyewitness accounts. Their pattern of results may be a consequence of confusion between incorrect memory and reality when both share similar perceptual details (Maras and Bowler, 2010).

The correlations with executive tasks provide converging evidence for the contribution of attention regulation to memory performance in our ASD participants. First, fluency correlated with priming effects after global processing suggesting that additional attentional strategies may be implicated, enabling participants to go beyond details and process items globally. Second, in the explicit memory task, attentional dysfunction may serve to diminish accuracy in the local condition, i.e., by diminishing correct recognitions and increasing false recognition rates. Once more, when individuals with ASD are instructed to process certain stimulus details, attenuated attention disengagement may prevent the processing of sufficient other information needed to create distinctive memory traces. These data are consistent with current accounts that focus on attentional mechanisms (Kaldy et al., 2013).

There are other findings of the present study that also merit consideration. In both experiments individuals with ASD benefitted from global processing either in implicit or explicit conditions. This advantage may be more limited for implicit and time-limited compared to explicit memory tasks. These results are consistent with the hypothesis that individuals with ASD are able to use global strategies when the experimental design is appropriate (Mottron et al., 2006, 2013b) and when given enough time (Van der Hallen et al., 2015). These considerations raise important issues for interventions. The evidence from the present experiments suggests that if educators were to instruct individuals with ASD to orient their attention toward the whole and to give them more time to process material, they should perform as well as TD individuals. Conversely, orienting attention toward specific details may increase confusion between activities that share similar details.

## Conclusion

Enhanced perceptual abilities are a well-known clinical feature of ASD. However, there are no studies that have looked at the impact of this superiority for local processing on memory functioning. The major limitation of this work remains the limited size of our sample. However, the present preliminary data bring some arguments in favor of a significant effect of detail focus on both implicit and explicit memory. The study needs to be replicated with a large sample but already highlights the need to take into account atypical perception in some individuals with ASD (i.e., with local bias) in order better to understand memory functioning in a research setting, and to use more specific and appropriate instructions in educational settings.

In addition and given the significant variability that characterizes Autism, it would be interesting to pursue this work to identify individual factors that may influence this pattern of performance.

## Data Availability Statement

The raw data supporting the conclusions of this article will be made available by the authors, without undue reservation.

## Ethics Statement

The studies involving human participants were reviewed and approved by ethics committee (CQI) of the French Institute of Health and Medical Research, a methodological committee (CCTIRS) associated with the French Ministry of Higher Education and Research, and the French Data Protection Authority (CNIL). Written informed consent to participate in this study was provided by the participants’ legal guardian/next of kin.

## Author Contributions

KL and BG-G designed the study. JM, LB, AH-D, GM, FG, and EM collected the data. PC participated to the statistical analyses. KL and BG-G wrote the manuscript. DB, FB-B, FE, and J-MB provided substancial modification to the manuscript. All the authors contributed to the article and approved the submitted version.

## Conflict of Interest

The authors declare that the research was conducted in the absence of any commercial or financial relationships that could be construed as a potential conflict of interest.
